# Epidemiology of Rabies in Lesotho: The Importance of Routine Surveillance and Virus Characterization

**DOI:** 10.3390/tropicalmed2030030

**Published:** 2017-07-19

**Authors:** Andre Coetzer, Jessica Coertse, Mabusetsa Joseph Makalo, Marosi Molomo, Wanda Markotter, Louis Hendrik Nel

**Affiliations:** 1 Department of Microbiology and Plant Pathology, Faculty of Natural and Agricultural Sciences, University of Pretoria, 0001 Pretoria, South Africa; andre.coetzer@up.ac.za; 2 Global Alliance for Rabies Control SA NPC, Erasmus Forum A434, South Erasmus Rand, 0181 Pretoria, South Africa; 3 Centre for Viral Zoonoses, Department of Medical Virology, Faculty of Health Sciences, University of Pretoria, 0001 Pretoria, South Africa; jessica.coertse@up.ac.za (J.C.); Wanda.Markotter@up.ac.za (W.M.); 4 Department of Livestock Services, Ministry of Agriculture and Food Security, Private Bag A82, 100 Maseru, Lesotho; mabusetsa1930@gmail.com (M.J.M.); molomomarosi@gmail.com (M.M.)

**Keywords:** surveillance, rabies, diagnosis, southern Africa

## Abstract

Rabies is widespread throughout Africa and Asia, despite the fact that the control and elimination of this disease has been proven to be feasible. Lesotho, a small landlocked country surrounded by South Africa, has been known to be endemic for rabies since the 1980s but the epidemiology of the disease remains poorly understood due to limited sample submission, constrained diagnostic capabilities, and a lack of molecular epidemiological data. Considering the existing challenges experienced in Lesotho, we aimed to evaluate the direct, rapid immunohistochemical test (DRIT) as an alternative to the direct fluorescent antibody (DFA) test for rabies diagnosis in Lesotho. Towards this aim, extensive training on the implementation and interpretation of the DRIT was hosted in Lesotho in April 2016 before both tests were applied to all samples subjected to routine rabies diagnosis at the Central Veterinary Laboratory (CVL). We found agreement between the DFA and DRIT assays in 90/96 samples (93.75%). The samples that produced inconsistent results (*n* = 6) were re-tested a further two times with both assays before being subjected to a real-time qPCR to confirm the diagnosis. Additionally, a statistically significant three-fold increase in the average number of samples submitted per month was observed after the DRIT implementation started, following continuous rabies awareness initiatives amongst the animal health professionals in the country over a 12-month period (*p* = 0.0279). Partial G-L intergenic regions of selected rabies-positive samples (*n* = 21) were amplified, sequenced, and subjected to phylogenetic analyses. Molecular epidemiological analyses, which included viruses from neighbouring provinces in South Africa, suggested that at least three independent rabies cycles within Lesotho were implicated in instances of cross-border transmission. This study has evaluated alternative methods for diagnosing and improving rabies surveillance in Lesotho, as well as providing new information that would be of importance in the planning of future disease intervention campaigns, not only in Lesotho, but also in neighbouring South Africa.

## 1. Introduction

Canine-mediated rabies, caused by rabies virus (RABV), is a neglected tropical disease that has the highest case-fatality rate of any known infectious disease, accounting for an estimated 59,000 human deaths every year globally [1]. The burden associated with the disease is highest in developing countries and is typically a scourge on the poorest people in low and middle income countries (LMICs) [1,2,3]. On the African continent, rabies causes an estimated 21,000 (36% of the global canine-mediated human rabies cases) deaths annually, with the number of infected animals being several magnitudes higher [1]. While the availability of post-exposure prophylaxis (PEP) prevents the onset of human rabies after an exposure, the most cost-effective control measure is the routine vaccination of the domestic dog populations [3]. Unfortunately, various social, economic, and political factors contribute to the inadequate control of canine-mediated rabies in endemic areas, with human rabies cases occurring as a result thereof [1,3,4].

Rabies was first recorded in Lesotho in the 1980s when the disease was introduced into the northeastern part of the country from the KwaZulu-Natal (KZN) province of South Africa [5]. Subsequent to the introduction, rabies spread throughout the country within two years and has remained endemic ever since [5,6,7,8]. Despite annual dog vaccination campaigns undertaken throughout the country, the control and elimination of rabies in the country is complicated by a low estimated vaccination coverage [8] and the fact that Lesotho is bordered by three rabies-endemic South African provinces, viz. the Free State (FS), Eastern Cape (EC),and KZN provinces. These provinces have historically been shown to contribute to the cross-border transmission of rabies between the two countries [5,9,10,11].

The transboundary spread of rabies between South Africa and Lesotho was first confirmed during a molecular epidemiological investigation of canine rabies in the FS province [9]. During that study, 13 sequences originating from Lesotho were included in the molecular epidemiological analyses. It was not only shown that the FS province and Lesotho shared the same epidemiological cycle, but historical surveillance data also indicated that canine rabies was most likely introduced into the FS province after crossing the border from Lesotho [9]. Since then, no further molecular epidemiological studies had been performed in either the FS province or Lesotho, limiting the contemporary information needed to guide disease intervention campaigns within the two countries.

**Figure 1:**
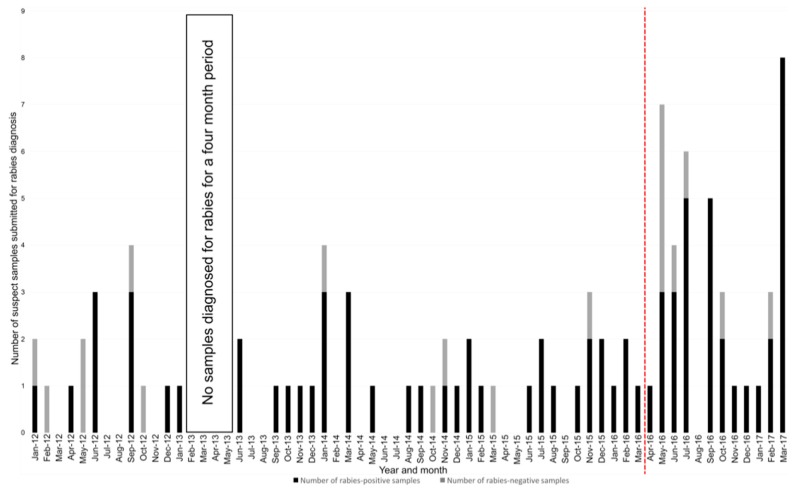
The number of positive and negative samples submitted per month for rabies diagnosis at the Central Veterinary Laboratory in Maseru, Lesotho (2012–2016). The number of samples subjected to rabies diagnosis per month between January 2012 and March 2017 are depicted as vertical bars. The number of rabies-positive samples are depicted as the black-filled bars, while the number of rabies-negative samples are depicted as the grey-filled bars. Months where no vertical bars are present indicate a month where no samples were subjected to rabies diagnosis. The vertical dashed line represents the start of the twelve-month study period during which the DRIT was actively promoted in the country.

One of the main limitations to the improved epidemiological understanding of rabies in Lesotho is limited surveillance data. Although rabies is a notifiable disease in Lesotho, the surveillance network has historically been constrained by a lack of samples subjected to routine rabies diagnosis at the only laboratory capable of diagnosing rabies in the country, the Central Veterinary Laboratory (CVL) in Maseru (Figure 1). The limited surveillance data and molecular epidemiological information directly contributed to the underestimated burden of the disease [8], as well as the subsequently low prioritisation and lack of rabies control activities.

In an effort to enhance the rabies diagnostic capabilities and capacity in Lesotho, we implemented the direct, rapid immunohistochemical test (DRIT) in a twinning approach to the gold standard direct fluorescent antibody (DFA) test at the CVL in Maseru. Throughout this project, the routine submission of samples was continuously encouraged amongst the animal health professionals operating within the country. The awareness activities not only facilitated the submission of samples to be tested with the DFA and DRIT assays, but also enabled us to investigate whether stimulating the members of the expert network into collecting and submitting samples would result in a statistically-significant increase in sample submission over a 12-month period. To improve molecular epidemiological information within Lesotho, a sub-set of the samples—including viruses from the neighbouring provinces of South Africa—was analysed by targeting the G-L intergenic region of RABV cDNA.

## 2. Materials and Methods

### 2.1. Diagnostic Training and Sample Cohort

Before the routine implementation of the DRIT assay in Lesotho, a workshop on the implementation and interpretation of the assay was presented in April 2016 by a South African diagnostician experienced in the use and interpretation of the assay [12]. Over the course of the five-day workshop held at the CVL in Maseru, four local diagnosticians were introduced to the DRIT assay and its use in a diagnostic setting by applying the assay blindly to a cohort of archival samples (*n* = 57) that had been found to be either rabies-positive or -negative with the gold standard DFA test. The DFA test applied at the CVL in Lesotho relied on a FITC-labelled polyclonal antibody (PAb) preparation prepared by the Agricultural Research Council-Onderstepoort Veterinary Institute (ARC-OVI, Rabies Division) prior to the samples being stored in a 50% glycerol-saline solution at ambient temperature (Table 1) [13,14]. While all of the diagnosticians were familiar with the DFA, the samples in this study were the first DRIT-diagnosed samples that the local diagnosticians had interpreted.

After the diagnostic training programme had been completed in April 2016, the DRIT and DFA assays were routinely applied concurrently to newly-submitted brain tissue samples (*n* = 39) that were collected by the trained technicians without further supervision from the DRIT trainer (Table 1). Additionally, the expert network of trained animal technicians and veterinarians (both state and private) operating within Lesotho were continuously contacted via short messages service (SMS), emails or telephonic calls and encouraged to collect samples for diagnostic confirmation within their communities. The aforementioned messages were sent on a monthly basis to the animal health technicians responsible for each of the ten districts, who subsequently disseminated the information to all of relevant stakeholders operating in the specific resource centres. Suspect samples were subsequently collected, submitted, and diagnosed in support of the project which, in turn, increased the level of surveillance data generated for the country.

### 2.2. Direct, Rapid Immunohistochemical Test (DRIT)

All of the suspect rabies samples diagnosed in this study (*n* = 96) were subjected to the DRIT diagnostic assay according to the standard operating procedure [15,16]. The DRIT assay used a biotinylated anti-ribonucleoprotein PAb preparation prepared by the ARC-OVI, Rabies Division, and both positive and negative controls, consisting of homogenized brain material that had been confirmed as rabies-positive and -negative, respectively, by the OIE Rabies Reference Laboratory in South Africa, were included in every run.

Briefly, the brain material was homogenised prior to diagnostic confirmation to prevent viral tissue tropism from influencing the outcome of the DRIT test. A single touch impression was made from the brain tissues by placing a small amount of homogenised material on clean tissue paper. Touch impressions of the samples were allowed to air dry for five minutes before being submerged in 10% neutral buffered formalin (Sigma-Aldrich, Missouri, MO, USA) for ten minutes. After fixation, the touch impressions were re-hydrated by dip-rinsing the slides in TRIS phosphate-buffered saline (TPBS) buffer (phosphate-buffered saline (PBS), pH of 7.5 (Whitehead Scientific, Stikland, South Africa) containing 1% Tween 80 (Merck, Kenilworth, NJ, USA) and, thereafter, were submerged in 3% hydrogen peroxide (Merck) for ten minutes at room temperature in order to halt all endogenous peroxidase activity. Subsequent to the hydrogen peroxide flooding, the slides were dip-rinsed in fresh TPBS buffer and the excess buffer was shaken from the slides. The areas surrounding the smear impressions were blotted using a fresh paper towel. Anti-ribonucleoprotein biotinylated polyclonal antibody (PAb) preparation (1:300 working concentration) was applied dropwise until the impression was completely covered. After the application of the antibody, the slides were placed in a humidity chamber and incubated at room temperature for ten minutes, and subsequently dip-rinsed in fresh TPBS buffer. The excess buffer was shaken from the slides, and the areas surrounding the smear impressions were blotted using a fresh paper towel. All of the touch impressions were covered in a ready-to-use solution of 2 μg/mL streptavidin-peroxidase (Kirkegaard and Perry Laboratories, Gaithersburg, MD, USA), after which the slides were transferred to a humidity chamber. The humidity chamber was incubated at room temperature for ten minutes, and the slides were dip-rinsed in fresh TPBS buffer. The excess buffer was shaken from the slides and the areas surrounding the smear impressions were blotted using a fresh paper towel. The impressions were covered in a working solution of the 3-amino-9-ethylcarbazole (AEC) chromogen (Sigma-Aldrich), and the slides were transferred to a humidity chamber and incubated at room temperature for five minutes. After sufficient staining had occurred, the slides were submerged in distilled water. The touch impressions were counterstained with a 1:2 dilution of Gill’s formulation #2 (Sigma-Aldrich) for two minutes before they were dip-rinsed in distilled water in order to wash away the residual counterstain. Finally, the slides were mounted with a water-soluble mounting medium (1×PBS (Whitehead Scientific)/glycerol (Sigma-Aldrich) prepared 1:1) and examined by light microscopy (Olympus, CX21) at both 200× and 400× magnification in order to score the respective immunoreactivity based on both the presence and staining intensity of the visible red inclusions present on the blue cellular background [16].

### 2.3. Direct Fluorescent Antibody (DFA) Test

The DFA diagnostic assay was repeated on all the archival samples that produced discrepant results from those obtained after performing the DRIT assay during the training programme and, thereafter, in parallel to the DRIT assay on all of the samples that arrived for routine rabies diagnosis. The DFA test was performed according to the standard operating procedure [13,17] and relied on treating homogenised tissue impressions with a 1:1000 working dilution of FITC-labelled anti-ribonucleoprotein PAb preparation (ARC-OVI, Rabies Division, Pretoria, South Africa) in order to confirm any false results. Two microscopists based at the CVL interpreted the results on a Zeiss Axiovert 25 (Axiolab) fluorescent microscope at a magnification of 400× (excitation: 490 nm; Emission: 525 nm) in a blind reading in order to eliminate reader bias.

### 2.4. Resolving Diagnostic Incongruities

Samples that produced diagnostic incongruities observed between the DFA and DRIT assays (*n* = 6) were first re-tested a further two times with both assays at the CVL to confirm the relevant diagnostic results. Continued discrepancies were resolved by the attempted amplification of viral nucleic acid from the total RNA extracted from each sample (Table 1). Briefly, the total RNA of the homogenised brain tissue samples (*n* = 6) was extracted using the Direct-Zol™ RNA MiniPrep Kit (Zymo Research, Irvine, CA, USA) as per the manufacturer’s instructions. An established “one-step” quantification real-time PCR (qRT-PCR) assay targeting the partial nucleoprotein gene was applied to amplify any lyssavirus RNA present in the brain material [18].

### 2.5. Data Analysis

The determination of the diagnostic sensitivity, specificity and respective confidence intervals of the DFA and DRIT diagnostic assays was determined using an exact binomial distribution (MedCalc 12.2.1.0, Ostend, Belgium). In order to determine whether the increase in sample submissions observed during the study period was statistically significant, a one-way analysis of variation (ANOVA) analysis was performed using the Epi Info™ software (version 7.2). The one-way ANOVA analysis was used across differences in mean sample submissions per month prior to the onset of the study (January 2012–March 2016) and within the study period (April 2016–March 2017).

### 2.6. Molecular Epidemiology of Rabies in LESOTHO

#### 2.6.1. Sample Cohort

Twenty-one rabies-positive brain samples, collected and stored at the CVL in Maseru, were selected and used for molecular epidemiological characterization and phylogenetic analysis (Table S1). The representative panel of samples from across the country was chosen based on both the location where the samples were originally collected within Lesotho and the amount of available brain material stored at the CVL.

#### 2.6.2. Viral RNA Extractions, PCR, and Sequencing

The total RNA of all of the brain tissue samples (*n* = 21) was extracted using the Direct-Zol™ RNA MiniPrep Kit (Zymo Research). A reverse transcription PCR was performed on the rabies-positive samples using the G(+) and L(−) primers [19,20], which amplifies the cytoplasmic domain of the glycoprotein gene and the adjacent G-L intergenic region of the RABV genome.

The PCR-positive products obtained from the 21 samples were electrophoresed on a standard 1% agarose gel and subsequently gel-extracted and purified using the Zymocelan™ Gel DNA Recovery kit per the manufacturer’s instructions (Zymo Research). Both the forward and reverse strands of the purified PCR amplicons were sequenced using the respective PCR primers and the BigDye^®^ Terminator v3.1 sequencing reaction kit per the manufacturer’s instructions (Applied Biosystems). The Sanger sequencing was performed using an ABI 3100 automated capillary sequencer situated at the University of Pretoria, RSA. Consensus sequences were generated and trimmed to 592 nucleotides (nt), representing the cytoplasmic domain of the glycoprotein gene and the adjacent G-L intergenic region of the RABV genome using the CLC Main Workbench software (CLC Bio, Version 7.7.2). The final sequences were subsequently submitted to the NCBI GenBank and allocated unique accession numbers (MF197287–MF197307) (Table S1).

#### 2.6.3. Phylogenetic Analysis

The phylogenetic analysis included a total of 100 G-L intergenic region sequences obtained from Lesotho and selected neighbouring South African provinces (Table S1). An alignment of the collection of sequences was created using the ClustalW subroutine of the BioEdit software [21] and the phylogenetic analysis was performed using a Bayesian Markov Chain Monte Carlo (MCMC) method in the BEAST software package (version 1.8.1) [22]. The best fitting DNA substitution model (TVMef+G) was selected using the Akaike’s information criterion (AIC) determined using the JModel software (version 2.1.3). For the purpose of the phylogenetic analysis, three independent Markov chains were sampled for 50 million states and a sampling frequency of 50,000 was combined after discarding at least 10 per cent burn. The posterior distributions were inspected using Tracer (version 1.6) to ensure adequate mixing and convergence. The associated statistics were summarised as a maximum clade credibility tree and visualised using the FigTree software (version 1.4.2).

## 3. Results

### 3.1. Statistical Analysis of DRIT Diagnostic Efficacy

The number of true-positive (*n* = 72) and negative (*n* = 24) samples (determined by either agreeing DRIT and DFA assays or qRT-PCR amplification of nucleic acid) were used to determine the diagnostic efficacy of the DFA and DRIT assays (Table 2). Based on the confirmatory results provided by the “one-step” quantification qRT-PCR assay, the DFA test had produced an inaccurate result in six archival brain samples (DFA-positive but DRIT- and qRT-PCR-negative), indicating a reduced diagnostic specificity of 75% (Table 2). In contrast, the DRIT and qRT-PCR produced identical results for the samples in question and the DRIT was considered to have an overall diagnostic sensitivity and specificity of 100% (Table 2).

### 3.2. Increased Sample Submission and Distribution Analysis

Over a 51-month period prior to the start of the DRIT implementation in Lesotho (January 2012–March 2016), the CVL had intermittently received and diagnosed an average of one sample per month. Over the same 51-month period, a total of 17 months (33%) had passed without any samples being submitted for rabies diagnosis (Figure 1). During the first 12 months of DRIT implementation in Lesotho (April 2016–March 2017), the average number of samples had increased to three samples per month, primarily due to the continued encouragement of the trained animal health professionals, resulting in a statistically significant three-fold increase in the monthly sample submission for rabies diagnosis (*p* = 0.0279, 95% CI: 0.2449 to 3.4556) (Figure 1). The significance of the increase is further appreciated when considering that samples were submitted regularly for every month of the year, with the exception of August 2016.

### 3.3. Molecular Epidemiology

**Figure 2:**
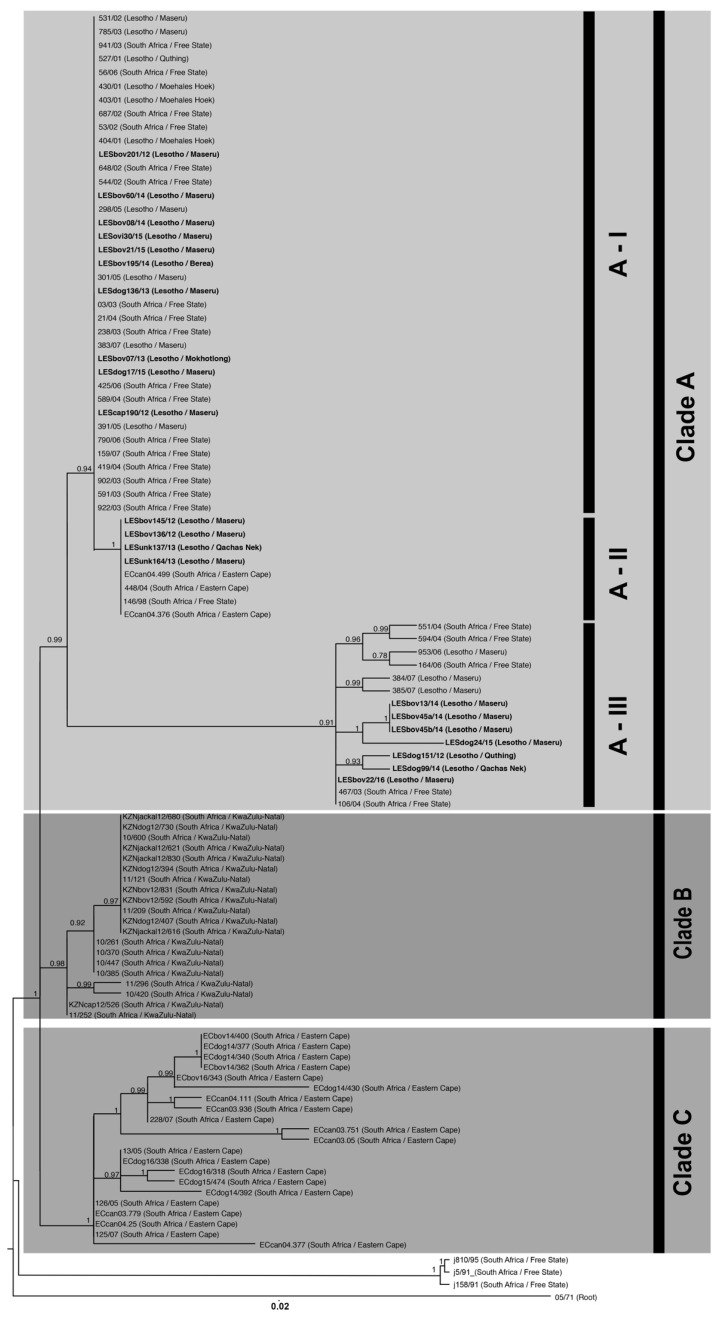
Maximum clade credibility tree of the cytoplasmic domain of the G-L intergenic region of RABV sequences originating from selected sub-Saharan African countries (Table S1). The horizontal branch lengths are proportional to the similarity of the sequences within and between groups and all branches with a posterior probability of 0.75 or less were collapsed. A bat-eared fox sequence from the Western Cape Province (isolate 05/71) was used to root the tree. The new sequences generated in this study have been indicated in a bold font (Table S1).

**Figure 3:**
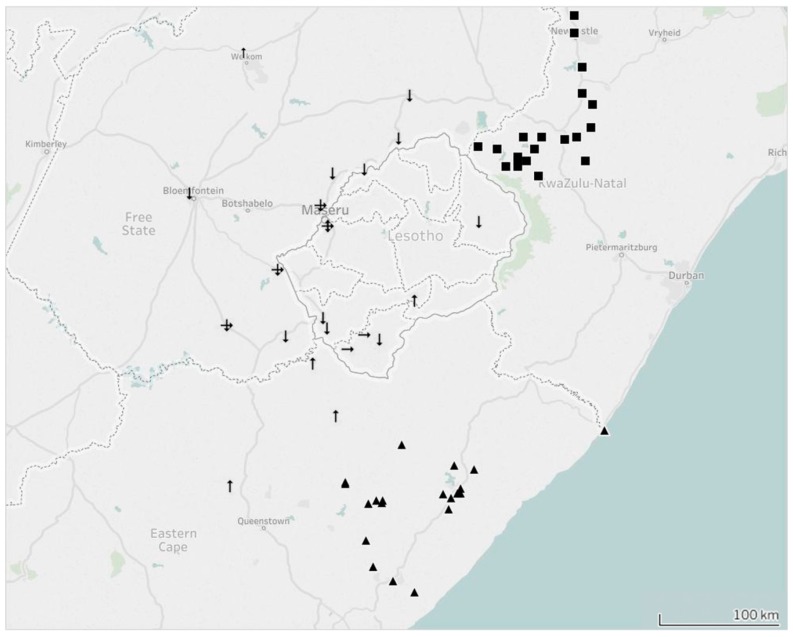
Illustrated map showing the inferred cross-border spread of endemic dog rabies between Lesotho and South Africa. Samples forming part of Clade A have been indicated with arrows, Clade B with squares, and Clade C with triangles. The three sub-clades have been indicated as follows: arrows facing downwards (sub-clade AI), arrows facing upwards (sub-clade AII) and arrows facing to the right (sub-clade AIII).

The RABV sequences included in this study could, phylogenetically, be divided into three clades (Clade A–C), with each clade comprising separate lineages with posterior probability scores of 0.99, 0.96 and 1, respectively, (Figure 2). Clade A consisted largely of canid rabies viruses (*n* = 79) from Lesotho and two provinces of South Africa (the FS province and the northern region of the EC province) (Figure 3). Clade B consisted primarily of rabies-positive samples collected from the north-eastern region of the KZN province where a sylvatic outbreak is currently ongoing (2012–2017) [23] (Figure 3). The sequences that form part of Clade B clustered independently from Clade A as the cross-border spread of rabies between Lesotho and the KZN province is limited to a large extent by geographical barriers such as the Drakensberg and Maloti mountain ranges, which limit the movement of people and animals between Lesotho and the neighbouring South African province to the south [9]. Clade C was comprised of rabies-positive samples collected from the southeastern and central regions of the EC province bordering the KZN province (Figure 3).

Clade A could be further divided into three separate sub-clades (sub-clade AI–AIII) with each sub-clade representing independent endemic cycles that are both geographically defined and shared with its immediate regional neighbour (Figure 2). Sub-clade AI consisted exclusively of RABV sequences obtained from the FS province and Lesotho (Figure 3). The clustering observed in this sub-clade is similar to the cross-border spread that was observed in an earlier study investigating the increased number of dog-rabies cases in the FS province [9]. The branching clusters observed within the sub-clade illustrated that the viruses in this clade were closely related, which suggests that a single RABV strain historically circulated between Lesotho and the FS province. This finding was also in line with the previous findings where the authors concluded that canine rabies most likely spread from Lesotho to the FS prior to becoming an established active cycle spanning the entire region [9].

Sub-clade AII comprised of a single RABV sequence (146/98) collected from a dog in the FS province in 1998 and RABV sequences obtained from Lesotho and the northern region of the EC province (Figure 3). Evidence of an endemic cycle between the FS and northern region of the EC province had previously been suggested, but our study provides evidence that rabies virus-positive samples, identified in Lesotho between 2012 and 2013, can also be linked to this cycle [9].

The third clade, sub-clade AIII, highlighted genetic relatedness between isolates originating from the capital, Maseru, the rural areas in the southernmost district of Lesotho (Quthing) and the FS province of South Africa (Figure 3). To our knowledge, this is the first report of an independent phylogenetic relationship between rabies viruses from the FS province and this specific region of Lesotho.

## 4. Discussion

In this study, we analysed 96 samples and found that 93.75% had correctly been diagnosed as either rabies-positive or -negative using the DFA assay when compared with qRT-PCR results. Several reasons could explain the occurrence of the false-positive results associated solely with the archival samples, especially when considering that the DFA test had been implemented in a resource-limited laboratory, where the maintenance of equipment is often lacking [24,25]. The most common possible reasons for incongruent results, viz. antigen degradation/putrefaction and the misinterpretation of fluorescence when performing the DFA test, were all considered [12,26,27,28,29,30]. However, sample degradation should be discussed in more detail. During our investigation, the archival samples had been placed in 50% glycerol-saline solution upon collection, before being stored at ambient temperature for extended periods of times (collected between 2011 and 2016). Despite relying on the glycerol-saline solution to preserve the samples, some of the samples had visible and olfactory signs of putrefaction (varying between samples) when tested with the DRIT assay.

Samples that have undergone putrefaction due to inadequate sample storage could contain bacterial or fungal contamination, which could, in turn, produce a large amount of non-specific fluorescence [27]. The presence of non-specific fluorescence could be misinterpreted as positive signals in the hands of an inexperienced reader or a reader relying on an improperly calibrated fluorescence microscope, leading to false-positive results being recorded [12]. The use of sub-optimal equipment while relying on an inadequate infrastructure in developing countries, is commonplace and a genuine concern [12]. Regardless of the cause of the incorrect diagnostic outcomes, the results reported here do not reflect negatively on the competency of the CVL staff. Instead, the technical challenges and difficulties associated with equipment, cold storage and the shipment of samples in developing countries, are highlighted.

This study adds to the realization of the versatility of the DRIT assay. In our view, this test should be considered for routine rabies diagnosis as it has been shown to be advantageous to resource-limited settings such as the one described here. Apart from having a diagnostic efficacy that is comparable to that of the DFA test, the DRIT is less sensitive to potential misinterpretations of results by not relying on fluorescent signals [12,24,31,32,33]. By reducing the visible interfering background staining (especially in samples that have undergone putrefaction), and simplifying the differentiation between a positive and negative result, the DRIT provides accurate diagnostic results by adequately-trained personnel.

In this study, 62/96 (65%) of the samples diagnosed as rabies-positive originated from the Maseru district where the CVL is located, with the number of samples dissipating as the districts locate further away from the CVL (Table 1). The positive correlation between the number of samples submitted for routine rabies diagnosis and the distance from the CVL indicates that the surveillance network could be enhanced even further by decreasing the distance samples must travel before reaching a laboratory. Considering the financial benefits associated with starting and routinely implementing the DRIT test at new diagnostic facilities, the assay could be used for decentralised rabies diagnosis without incurring a large capital investment [15]. In recognition of this fact, two decentralised diagnostic facilities in the Mokhotlong and Moehale’s Hoek districts of Lesotho have already been identified as possible locations for decentralised routine rabies diagnosis in the future.

The submission and diagnosis of suspect samples provide a basic burden indicator for rabies, but in situations as described here, the resolution of the surveillance network remains very limited. Molecular epidemiological analyses are helpful in advancing our understanding of virus cycles and the transboundary movement of viruses in the larger region. The phylogenetic component of this study represents the first application of molecular epidemiology to specifically investigate the occurrence of endemic rabies within Lesotho. The results of our study were helpful to demonstrate that host species in Lesotho maintain three independent rabies cycles, which could impact negatively on any rabies intervention initiatives that are implemented in either of the two countries as animals can move freely between the two countries due to the porous nature of the borders on the African continent [12,34].

Although the presence of cross-border spread of rabies between Lesotho and the FS province is not a new observation [9], the sequence similarity observed for samples originating from Lesotho and the FS province (sub-clade AI) indicates that cross-border spread has been ongoing since the last investigation was performed in 2009 (Figure 2). As such, it could be speculated that human movement (along with their livestock and companion animals) is responsible for the ongoing exchange of rabies between two geographically-separated dog populations. This supposition is further supported by the observation that the Maseru district, which is approximately 11 km from the nearest border post between South Africa and Lesotho, is a potential rabies hotspot where all the various known genetic cycles of rabies occur (sub-clade AI–AIII) (Figure 3).

The sequence similarity observed for samples forming part of sub-clade AII indicates that cross-border spread occurs between Lesotho and two neighbouring provinces of South Africa (EC and FS provinces) (Figure 2). To our knowledge, the involvement of Lesotho in this active cycle is a novel observation and contradicts previous speculations that the sequence similarity between samples collected in the FS and EC province was due to a single spill-over event [9].

Sub-clade AIII demonstrates the value of undertaking continuous molecular investigations while relying on contemporary samples. Prior to this study, no molecular epidemiological information was available for this rural area in the southern highland districts of Lesotho. By including samples recently collected within Lesotho, it has become evident that rabies is not only present in this region, but that the Lesotho samples in this sub-clade have a higher level of sequence diversity to those in the other sub-clades (Figure 2). Our conjecture is that the endemic cycle observed in sub-clade AIII might be maintained by sylvatic species such as the black-backed jackal rather than dog populations. This observation is based on the sequence diversity, high number of rabies-positive livestock cases, comparatively low number of rabies-positive dog cases from the area, and the presence of jackal species in the surrounding highlands areas of Lesotho [35,36,37]. However, the lack of samples originating from sylvatic species in Lesotho prevents any firm conclusion in this regard.

## 5. Conclusions

The presence of multiple endemic cycles involved in the cross-border spread of rabies between Lesotho and South Africa highlight the importance of regional collaboration towards rabies control [38,39]. Without the regional collaboration of all the stakeholders, the success of disease intervention campaigns will not be sustainable or long-lasting as the re-introduction of immunologically naïve dog populations could result in a reduced vaccination coverage or negative impact on the sustainability of existing vaccination campaigns. By creating vaccination buffer zones on both sides of pre-determined national or regional borders [40], the re-introduction of rabies can be prevented and the vaccination coverage can be systematically increased and maintained within and beyond the buffer zones. As such, the collaboration between all interested stakeholders will not only prevent low vaccination coverage, but will also ensure that neighbouring countries work towards the same objective of being declared free of canine-mediated human rabies by 2030, an objective in line with the sustainable development goals [41].

## Figures and Tables

**Table 1:** Neuronal tissue sample cohort from Lesotho depicting the initial diagnostic results from the CVL in Maseru, Lesotho, the diagnostic discrepancies and their independent molecular confirmation at the laboratory in South Africa.

	Sample Number	Species	Town	DRIT Result	DFA Result	Real-Time PCR Result
1 *	15/09/11	Bovine	Maseru	Positive	Positive	---
2 *	254/09/11	Bovine	Mokhotlong	Negative	Negative	---
3 *	11/01/12	Bovine	Berea	Positive	Positive	---
4 *	24/01/12	Bovine	Maseru	Negative	Negative	---
5 *	04/02/12	Bovine	Maseru	Negative	Negative	---
6 *	106/04/12	Bovine	Maseru	Negative	Negative	---
7 *	113/05/12	Bovine	Maseru	Negative	Negative	---
8 *	123/05/12	Canine	Maseru	Negative	Negative	---
9 *	14/06/12	Canine	Maseru	Positive	Positive	---
10 *^,^#	145/06/12	Bovine	Maseru	Positive	Positive	---
11 *^,^#	136/06/12	Canine	Maseru	Positive	Positive	---
12 *	12/09/12	Bovine	Maseru	Negative	Negative	---
13 *^,^#	151/09/12	Bovine	Quthing	Positive	Positive	---
14 *^,^#	190/09/12	Canine	Maseru	Positive	Positive	---
15 *^,^#	201/09/12	Caprine	Maseru	Positive	Positive	---
16 *	211/10/12	Canine	Maseru	Positive	Positive	---
17 *	276/12/12	Bovine	Maseru	Positive	Positive	---
18 *^,^#	07/01/13	Bovine	Mokhotlong	Positive	Positive	---
19 *^,^#	136/06/13	Bovine	Maseru	Positive	Positive	---
20 *^,^#	137/06/13	Canine	Qacha’s Nek	Positive	Positive	---
21 *^,^#	164/09/13	Canine	Maseru	Positive	Positive	---
22 *	10/10/13	Bovine	Berea	Positive	Positive	---
23 *	05/11/13	Bovine	Berea	Positive	Positive	---
24 *^,^#	194/12/13	Canine	Maseru	Positive	Positive	---
25 *^,^#	08/01/14	Bovine	Maseru	Positive	Positive	---
26 *	10/01/14	Bovine	Maseru	Negative	Negative	---
27 *^,^#	13/01/14	Bovine	Maseru	Positive	Positive	---
28 *	23/01/14	Canine	Maseru	Positive	Positive	---
29 *	20/03/14	Bovine	Maseru	Positive	Positive	---
30 *^,^#	45a/03/14	Bovine	Maseru	Positive	Positive	---
31 *^,^#	45b/03/14	Bovine	Maseru	Positive	Positive	---
32 *^,^#	60/05/14	Canine	Qacha’s Nek	Positive	Positive	---
33 *	26/08/14	Bovine	Maseru	Positive	Positive	---
34 *^,^#	99/09/14	Bovine	Maseru	Positive	Positive	---
35 *	21/10/14	Canine	Maseru	Negative	Negative	---
36 *	192/11/14	Bovine	Berea	Negative	Negative	---
37 *	193/11/14	Canine	Qacha’s Nek	Positive	Positive	---
38 *	10/12/14	Equine	Berea	Negative °	Positive °	Negative
39 *	07/01/15	Canine	Maseru	Positive	Positive	---
40 *^,^#	30/01/15	Ovine	Maseru	Positive	Positive	---
41 *^,^#	17/02/15	Canine	Maseru	Positive	Positive	---
42 *	18/03/15	Feline	Maseru	Negative	Negative	---
43 *	18/06/15	Ovine	Qacha’s Nek	Positive	Positive	---
44 *	150/11/15	Canine	Maseru	Positive	Positive	---
45 *^,^#	21/07/15	Bovine	Maseru	Positive	Positive	---
46 *	23/07/15	Bovine	Maseru	Positive	Positive	---
47 *^,^#	24/08/15	Canine	Maseru	Positive	Positive	---
48 *	06/10/15	Canine	Maseru	Positive	Positive	---
49 *	10/11/15	Caprine	Maseru	Positive	Positive	---
50 *	26/11/15	Bovine	Mohales Hoek	Negative °	Positive °	Negative
51 *	149/11/15	Caprine	Maseru	Positive	Positive	---
52 *	29/12/15	Canine	Maseru	Negative °	Positive °	Negative
53 *	161/12/15	Canine	Berea	Positive	Positive	---
54 *	14/01/16	Canine	Maseru	Negative °	Positive °	Negative
55 *#	22/02/16	Bovine	Maseru	Positive	Positive	---
56 *	26/02/16	Bovine	Berea	Negative °	Positive °	Negative
57 *	03/03/16	Canine	Maseru	Negative °	Positive °	Negative
58 +	95/04/16	Bovine	Berea	Positive	Positive	---
59 +	100/05/2016	Canine	Maseru	Negative	Negative	---
60 +	105/05/2016	Canine	Berea	Negative	Negative	---
61 +	109/05/2016	Canine	Maseru	Positive	Positive	---
62 +	110/05/2016	Canine	Berea	Positive	Positive	---
63 +	113/05/2016	Ovine	Berea	Negative	Negative	---
64 +	115/05/2016	Bovine	Berea	Positive	Positive	---
65 +	123/6/2016	Canine	Maseru	Positive	Positive	---
66 +	125/6/2016	Ovine	Maseru	Positive	Positive	---
67 +	127/06/2016	Porcine	Mohales Hoek	Negative	Negative	---
68 +	128/06/2016	Canine	Mohales Hoek	Positive	Positive	---
69 +	131/07/2016	Canine	Berea	Positive	Positive	---
70 +	132/07/2016	Canine	Maseru	Positive	Positive	---
71 +	133/07/2016	Canine	Maseru	Positive	Positive	---
72 +	136/07/2016	equine	Maseru	Negative	Negative	---
73 +	137/07/2016	Canine	Mohales Hoek	Positive	Positive	---
74 +	138/07/2016	Bovine	Mohales Hoek	Positive	Positive	---
75 +	163/09/2016	Ovine	Maseru	Positive	Positive	---
76 +	179/09/2016	Bovine	Maseru	Positive	Positive	---
77 +	182/09/2016	Canine	Mohales Hoek	Positive	Positive	---
78 +	184/09/2016	Canine	Maseru	Positive	Positive	---
79 +	185/09/2016	Canine	Maseru	Positive	Positive	---
80 +	196/10/2016	Canine	Berea	Positive	Positive	---
81 +	198/10/2016	Ovine	Maseru	Negative	Negative	---
82 +	199/10/2016	Bovine	Maseru	Positive	Positive	---
83 +	210/11/2016	Canine	Berea	Positive	Positive	---
84 +	234/12/2016	Canine	Maseru	Positive	Positive	---
85 +	30/01/2017	Bovine	Mafeteng	Positive	Positive	---
86 +	62/02/2017	Bovine	Maseru	Positive	Positive	---
87 +	63/02/2017	Canine	Maseru	Positive	Positive	---
88 +	71/02/2017	Bovine	Quthing	Negative	Negative	---
89 +	63/03/2017	Ovine	Maseru	Positive	Positive	---
90 +	69/03/2017	Bovine	Maseru	Positive	Positive	---
91 +	70/03/2017	Equine	Maseru	Positive	Positive	---
92 +	72/03/2017	Canine	Maseru	Positive	Positive	---
93 +	88/03/2017	Canine	Mokhotlong	Positive	Positive	---
94 +	89/03/2017	Bovine	Mokhotlong	Positive	Positive	---
95 +	90/03/2017	Canine	Mokhotlong	Positive	Positive	---
96 +	91/03/2017	Ovine	Mokhotlong	Positive	Positive	---

* denotes archival samples subjected to DRIT diagnosis during the diagnostic training programme; + denotes samples subjected to routine rabies diagnosis by both the DFA and DRIT assays; # denotes samples included in the molecular epidemiological analysis; The DFA and DRIT assays were repeated twice on samples that produced inconsistent results and the diagnostic outcomes of the two assays remained unchanged; **---** Real-time PCR not performed on samples without diagnostic incongruities.

**Table 2:** Diagnostic sensitivity, specificity of the direct rapid immunohistochemical test applied to a cohort of samples stored at the central veterinary laboratory in Lesotho.

	True Positive	False Positive	True Negative	False Negative	Diagnostic Sensitivity *	Diagnostic Specificity *
DFA	72	6	18	0	100% (95.01–100%)	75.00% (53.29–90.23%)
DRIT	72	0	24	0	100% (95.01–100%)	100% (85.75–100%)

* Values in brackets represented the 95% confidence interval (CI).

## Supplementary Materials

The following are available online at http://www.mdpi.com/2414-6366/2/3/30/s1, Table S1. Panel of rabies viruses from Lesotho and neighbouring South African provinces included in the phylogenetic analysis performed in this study.
