# Supplementary material for: Epidemiology of Rabies in Lesotho: The Importance of Routine Surveillance and Virus Characterization
Source: Trop Med Infect Dis. 2017 Jul 19;2(3):30. doi: 10.3390/tropicalmed2030030 (PMC6082089; doi:10.3390/tropicalmed2030030)
Supplement: Supplementary file 1 [file tropicalmed-02-00030-s001.pdf]

Supplementary Materials: Epidemiology of Rabies in Lesotho: The Importance of Routine Surveillance and Virus Characterization

Andre Coetzer, Jessica Coertse, Mabusetsa Joseph Makalo, Marosi Molomo, Wanda Markotter and Louis Hendrik Nel

Table S1. Panel of rabies viruses from Lesotho and neighbouring South African provinces included in the phylogenetic analysis performed in this study.

Sample Number	Species	Country	Province/District	Latitude #	Longitude#	Accession number
ECdog03.05	Canine	South Africa	Eastern Cape	–31.572065	28.80546	DQ841412
ECdog03.751	Canine	South Africa	Eastern Cape	–31.74376	28.68927	DQ841420
ECdog03.779	Canine	South Africa	Eastern Cape	–31.68712	28.04197	DQ841421
ECdog03.936	Canine	South Africa	Eastern Cape	–32.3323496	28.1446262	DQ841422
448/04	Canine	South Africa	Eastern Cape	–31.5532798	26.5550876	EU163315
ECdog04.25	Canine	South Africa	Eastern Cape	–31.6066561	28.8101537	DQ841405
ECdog04.111	Canine	South Africa	Eastern Cape	–32.4298987	28.3593437	DQ841409
ECdog04.376	Canine	South Africa	Eastern Cape	–30.524964	27.3645855	DQ841414
ECdog04.377	Canine	South Africa	Eastern Cape	–31.2084178	28.2351192	DQ841416
ECdog04.499	Canine	South Africa	Eastern Cape	–30.9690742	27.5906715	DQ841417
13/05	Canine	South Africa	Eastern Cape	–31.6066839	28.7780987	DQ431253
126/05	Human	South Africa	Eastern Cape	–32.2190338	27.9541894	GQ918295
125/07	Human	South Africa	Eastern Cape	–31.6727543	27.9834738	GQ918293

228/07	Human	South Africa	Eastern Cape	–31.6066839	28.7780987	GQ983417
ECdog14/340	Canine	South Africa	Eastern Cape	–31.66666667	28.05	MF197277
ECbov14/362	Bovine	South Africa	Eastern Cape	–31.53333333	27.68333333	MF197278
ECdog14/377	Canine	South Africa	Eastern Cape	–32	27.88333333	MF197279
ECdog14/392	Canine	South Africa	Eastern Cape	–31.65	28.71666667	MF197280
ECbov14/400	Bovine	South Africa	Eastern Cape	–31.51666667	27.68333333	MF197281
ECdog14/430	Canine	South Africa	Eastern Cape	–31.08333333	30.21666667	MF197282
ECdog15/474	Canine	South Africa	Eastern Cape	–31.38055556	28.74722222	MF197283
ECdog16/318	Canine	South Africa	Eastern Cape	–31.41111111	28.93888889	MF197284
ECdog16/338	Canine	South Africa	Eastern Cape	–31.61666667	28.63888889	MF197285
ECbov16/343	Bovine	South Africa	Eastern Cape	–31.69722222	27.90555556	MF197286
53/02	Canine	South Africa	Free State	–29.1860352	27.4438924	EU163343
544/02	Canine	South Africa	Free State	–28.9093365	27.5554547	EU163298
648/02	Canine	South Africa	Free State	–30.2949347	27.0970596	EU163364
687/02	Canine	South Africa	Free State	–29.7293819	27.0205594	EU163352
03/03	Canine	South Africa	Free State	–30.2015561	26.5255946	EU163310
238/03	Canine	South Africa	Free State	–30.2949347	27.0970596	EU163312

467/03	Canine	South Africa	Free State	–30.2015561	26.5255946	EU163350
591/03	Canine	South Africa	Free State	–29.1860352	27.4438924	EU163353
902/03	Canine	South Africa	Free State	–28.8777887	27.8663075	EU163332
922/03	Canine	South Africa	Free State	–29.085214	26.1595761	EU163333
941/03	Canine	South Africa	Free State	–28.8777887	27.8663075	EU163338
21/04	Canine	South Africa	Free State	–28.6154594	28.2000277	EU163329
106/04	Canine	South Africa	Free State	–29.7293819	27.0205594	EU163319
419/04	Canine	South Africa	Free State	–29.1860352	27.4438924	EU163314
551/04	Canine	South Africa	Free State	–29.7293819	27.0205594	EU163317
589/04	Canine	South Africa	Free State	–29.7293819	27.0205594	EU163377
594/04	Canine	South Africa	Free State	–29.7293819	27.0205594	EU163316
56/06	Canine	South Africa	Free State	–28.8777887	27.8663075	EU163360
164/06	Canine	South Africa	Free State	–29.1860352	27.4438924	EU163361
425/06	Canine	South Africa	Free State	–29.1860352	27.4438924	EU163356
790/06	Canine	South Africa	Free State	–28.2423025	28.3111262	EU163383
159/07	Canine	South Africa	Free State	–28.9093365	27.5554547	EU163379
10/261	Canine	South Africa	KwaZulu-Natal	–28.62	29.83	KC660228

10/370	Canine	South Africa	KwaZulu-Natal	–28.52	30.08	KC660165
10/385	Canine	South Africa	KwaZulu-Natal	–28.32	30.1	KC660262
10/420	Canine	South Africa	KwaZulu-Natal	–28	30	KC660195
10/447	Bovine	South Africa	KwaZulu-Natal	–28.6	29.94	KC660181
10/600	Canine	South Africa	KwaZulu-Natal	–28.6	29.42	KC660276
11/121	Canine	South Africa	KwaZulu-Natal	–28.8	30.03	KC660210
11/209	Bovine	South Africa	KwaZulu-Natal	–28.22	30	KC660206
11/252	Bovine	South Africa	KwaZulu-Natal	–27.55	29.92	KC660220
11/296	Canine	South Africa	KwaZulu-Natal	–27.7	29.92	KC660268
KZNdog12/394	Canine	South Africa	KwaZulu-Natal	–28.85	29.25	KY681361
KZNjackal12/680	Jackal	South Africa	KwaZulu-Natal	–28.7	29.53333333	KY681374
KZNbov12/592	Bovine	South Africa	KwaZulu-Natal	–28.8	29.36666667	KY681369
KZNcap12/526	Caprine	South Africa	KwaZulu-Natal	–28.85	29.36666667	KY681362
KZNbov12/831	Bovine	South Africa	KwaZulu-Natal	–28.93333333	29.56666667	KY681364
KZNjackal12/830	Jackal	South Africa	KwaZulu-Natal	–28.68333333	28.98333333	KY681371
KZNjackal12/621	Jackal	South Africa	KwaZulu-Natal	–28.76666667	29.36666667	KY681373
KZNjackal12/616	Jackal	South Africa	KwaZulu-Natal	–28.8	29.45	KY681365
KZNdog12/407	Canine	South Africa	KwaZulu-Natal	–28.7	29.16666639	KY681366
KZNdog12/730	Canine	South Africa	KwaZulu-Natal	–28.6	29.6	KY681367
403/01	Canine	Lesotho	Moehale's Hoek	–30.1425917	27.4673845	EU163386
404/01	Canine	Lesotho	Moehale's Hoek	–30.1425917	27.4673845	EU163385
430/01	Canine	Lesotho	Moehale's Hoek	–30.1425917	27.4673845	EU163392

527/01	Canine	Lesotho	Quthing	–30.3246496	28.0186087	EU163395
531/02	Canine	Lesotho	Maseru	–29.3632188	27.5143603	EU163391
785/03	Canine	Lesotho	Maseru	–29.3632188	27.5143603	EU163394
298/05	Canine	Lesotho	Maseru	–29.3632188	27.5143603	EU163393
301/05	Canine	Lesotho	Maseru	–29.3632188	27.5143603	EU163397
391/06	Canine	Lesotho	Maseru	–29.3632188	27.5143603	EU163398
953/06	Canine	Lesotho	Maseru	–29.3632188	27.5143603	EU163396
383/07	Canine	Lesotho	Maseru	–29.3632188	27.5143603	EU163399
384/07	Canine	Lesotho	Maseru	–29.3632188	27.5143603	EU163400
385/07	Canine	Lesotho	Maseru	–29.3632188	27.5143603	EU163401
LESbov201/12*	Bovine	Lesotho	Maseru	–29.3632188	27.5143603	MF197287
LEScap190/12*	Caprine	Lesotho	Maseru	–29.3632188	27.5143603	MF197287
LESdog151/12*	Canine	Lesotho	Quthing	–30.4015687	27.7080133	MF197287
LESbov145/12*	Bovine	Lesotho	Maseru	–29.3632188	27.5143603	MF197290
LESbov136/12*	Bovine	Lesotho	Maseru	–29.3632188	27.5143603	MF197291
LESunk137/13*	Unknown	Lesotho	Qacha's Nek	–29.9920147	28.3571523	MF197295
LESdog136/13*	Canine	Lesotho	Maseru	–29.3632188	27.5143603	MF197287

LESbov07/13*	Bovine	Lesotho	Mokhotlong	–29.3243263	28.9892494	MF197287
LESunk164/13*	Unknown	Lesotho	Maseru	–29.3632188	27.5143603	MF197287
LESdog99/14*	Canine	Lesotho	Qacha's Nek	–30.283402	27.866682	MF197296
LESbov60/14*	Bovine	Lesotho	Maseru	–29.3632188	27.5143603	MF197297
LESbov08/14*	Bovine	Lesotho	Maseru	–29.3632188	27.5143603	MF197298
LESbov195/14*	Bovine	Lesotho	Berea	–30.2271275	27.5055532	MF197299
LESbov13/14*	Bovine	Lesotho	Maseru	–29.3632188	27.5143603	MF197300
LESbov45a/14*	Bovine	Lesotho	Maseru	–29.3632188	27.5143603	MF197301
LESbov45b/14*	Bovine	Lesotho	Maseru	–29.3632188	27.5143603	MF197302
LESbov21/15*	Bovine	Lesotho	Maseru	–29.3632188	27.5143603	MF197303
LESdog24/15*	Canine	Lesotho	Maseru	–29.3632188	27.5143603	MF197304
LESdog17/15*	Canine	Lesotho	Maseru	–29.3632188	27.5143603	MF197305
LESovi30/15*	Ovine	Lesotho	Maseru	–29.3632188	27.5143603	MF197306
LESbov22/16*	Bovine	Lesotho	Maseru	–29.3632188	27.5143603	MF197307
05/71 (Phylogenetic tree root)	Bat-eared fox	South Africa	Western Cape	–31.6391218	18.5285072	DQ431385

* Denotes the samples that formed part of the sample cohort sequenced in this study.

# Approximate global positioning system (GPS) coordinates were used if the exact location of sampling was unknown.
